# Supplementary material for: Early-evening indoor and outdoor foraging by major malaria vectors in Nchelenge, Zambia
Source: PLOS Glob Public Health. 2026 Jul 27;6(7):e0005307. doi: 10.1371/journal.pgph.0005307 (PMC13405103; doi:10.1371/journal.pgph.0005307)
Supplement: S1 Table — This PCR uses the shared ITS2-A forward primer with species-specific reverse primers (RUF, 261 bp; MACU-R, 420 bp; and PRET-R, 422 bp). Each 25 µL PCR reaction consisted of 1x buffer, 1.0 mM dNTPs, 2 U of Taq polymerase, 50 pmol of each primer, and 1.0 µL extracted abdominal DNA. MultiGene OptiMax thermal cycler (Labnet International, Inc., Edison, New Jersey, USA) conditions consisted of an initial denaturation of 2 minutes at 95°C, followed by 35 cycles at 94°C for 30 seconds, 50°C for 30 seconds, and 72°C for 40 seconds. The final extension step was 72°C for 5 minutes. (DOCX) [file pgph.0005307.s001.docx]

**S1 Table. Conditions for maculipalpis/pretoriensis/rufipes species-specific PCR.**

| F Primer | F primer sequence | R primer | R Primer sequence | Amplicon size |
| --- | --- | --- | --- | --- |
| ITS2-A | 5’-TGTGAACTGCAGGACACAT-3’ | RUF-R | 5’-GTTCCTTGCACTATCGTAGCG-3’ | 261 |
|  |  | MACU-R | 5’-CGTGCCTAAGTGCTCACTAATG-3’ | 420 |
|  |  | PRET-R | ‘5-CCCGTTTATTAGGCGAGGACG-3’ | 422 |

This PCR uses the shared ITS2-A forward primer with species-specific reverse primers (RUF, 261 bp; MACU-R, 420 bp; and PRET-R, 422 bp). Each 25 µL PCR reaction consisted of 1x buffer, 1.0 mM dNTPs, 2 U of *Taq* polymerase, 50 pmol of each primer, and 1.0 µL extracted abdominal DNA. MultiGene OptiMax thermal cycler (Labnet International, Inc., Edison, New Jersey, USA) conditions consisted of an initial denaturation of 2 minutes at 95°C, followed by 35 cycles at 94°C for 30 seconds, 50°C for 30 seconds, and 72°C for 40 seconds. The final extension step was 72°C for 5 minutes.
