# Supplementary material for: Early-evening indoor and outdoor foraging by major malaria vectors in Nchelenge, Zambia
Source: PLOS Glob Public Health. 2026 Jul 27;6(7):e0005307. doi: 10.1371/journal.pgph.0005307 (PMC13405103; doi:10.1371/journal.pgph.0005307)
Supplement: S2 Table — * Household was visited by a test and treat program within the last year. + Household was visited by a filariasis MDA campaign within the last year (DOCX) [file pgph.0005307.s002.docx]

### **S2 Table. Household information.**

| **Variable** |  | **Inland** | **Lakeside** | **p-value** | |
| --- | --- | --- | --- | --- | --- |
| HOH age (mean (SD)) | | 52 (15.2) | 47.4 (8.9) | 0.43 |  |
| # people in HH (mean (SD)) | | 5.7 (2.9) | 7.1 (2.8) | 0.25 |  |
| # nets owned (mean (SD)) | | 1.5 (1) | 1.75 (1.1) | 0.57 |  |
| # rooms in HH (mean (SD)) | | 3.5 (0.9) | 4.9 (1.5) | 0.01 | ** |
| Distance to lake (km) (mean (SD)) | | 8.4 (3.9) | 0.6 (0.26) | <0.001 | *** |
| Distance to stream (km) (mean (SD)) | | 0.2 (0.15) | 2.2 (2.1) | 0.006 | *** |
| Distance from HH to animal pen (m) (mean (SD)) | | 5.9 (6.1) | 3.2 (3.4) | 0.19 |  |
| Roof materials (%) | | | | 0.04 | * |
|  | Thatch | 10 (41.7) | 4 (16.7) |  |  |
|  | Metal | 2 (8.3) | 8 (33.3) |  |  |
| IRS (%) | | | | 0.04 | * |
|  | Yes | 4 (16.7) | 10 (41.7) |  |  |
|  | No | 8 (33.3) | 2 (8.3) |  |  |
| Wall materials (%) | | | | 0.04 | * |
|  | Brick/concrete | 2 (8.3) | 8 (33.3) |  |  |
|  | Natural | 10 (41.7) | 4 (16.7) |  |  |
| Water source (%) | | | | 0.003 | *** |
|  | Borehole | 1 (4.2) | 9 (37.5) |  |  |
|  | Open well | 7 (29.2) | 1 (4.2) |  |  |
|  | Stream/pond | 4 (16.7) | 2 (8.3) |  |  |
| Eaves (%) | | | | 0.03 | * |
|  | Open | 10 (41.7) | 9 (37.5) |  |  |
|  | Closed | 2 (8.3) | 3 (12.5) |  |  |
| HOH occupation (%) | | | | 0.58 |  |
|  | Subsistence farmer | 11 (45.8) | 9 (37.5) |  |  |
|  | Other | 1 (4.2) | 3 (12.5) |  |  |
| Cooking materials (%) | | | | 0.15 |  |
|  | Wood | 4 (16.7) | 5 (20.8) |  |  |
|  | Coal/charcoal | 3 (12.5) | 6 (25) |  |  |
|  | Both | 5 (20.8) | 1 (4.2) |  |  |
| Owns goats (%) | | | | 0.58 |  |
|  | Yes | 9 (37.5) | 11 (45.8) |  |  |
|  | No | 3 (12.5) | 1 (4.2) |  |  |
| Owns pigs (%) | | | | 0.68 |  |
|  | Yes | 6 (25) | 4 (16.7) |  |  |
|  | No | 6 (25) | 8 (33.3) |  |  |
| Owns chickens (%) | | | | 1 |  |
|  | Yes | 8 (33.3) | 9 (37.5) |  |  |
|  | No | 4 (16.7) | 3 (12.5) |  |  |
| Owns dogs (%) | | | | 0.07 |  |
|  | Yes | 1 (4.2) | 6 (25) |  |  |
|  | No | 11 (45.8) | 6 (25) |  |  |
| Owns ducks (%) | | | | 0.31 |  |
|  | Yes | 1 (4.2) | 4 (16.7) |  |  |
|  | No | 11 (45.8) | 8 (33.3) |  |  |
| Owns cats (%) | | | | 0.58 |  |
|  | Yes | 3 (12.5) | 1 (4.2) |  |  |
|  | No | 9 (37.5) | 11 (45.8) |  |  |
| Malaria test and treat* (%) | | | | 0.21 |  |
|  | Yes | 7 (29.2) | 3 (12.5) |  |  |
|  | No | 5 (20.8) | 9 (37.5) |  |  |
| Filariasis MDA+ (%) | | | | 0.66 |  |
|  | Yes | 7 (29.2) | 9 (37.5) |  |  |
|  | No | 5 (20.8) | 3 (12.5) |  |  |
| * Household was visited by a test and treat program within the last year | | | | | |
| + Household was visited by a filariasis MDA campaign within the last year | | | | | |
