# Supplementary material for: Early-evening indoor and outdoor foraging by major malaria vectors in Nchelenge, Zambia
Source: PLOS Glob Public Health. 2026 Jul 27;6(7):e0005307. doi: 10.1371/journal.pgph.0005307 (PMC13405103; doi:10.1371/journal.pgph.0005307)
Supplement: S3 Table — *Overnight collections were only performed indoors for one replicate. (DOCX) [file pgph.0005307.s003.docx]

### **S3 Table. Molecular identification of species by trap type and location.**

| **Species** |  | | **Inland** | | |  | | **Lakeside** | | | **Total** |
| --- | --- | --- | --- | --- | --- | --- | --- | --- | --- | --- | --- |
|  | Indoor Early Evening | Indoor Overnight* | | Outdoor Gathering | Animal Pen | Indoor  Early Evening | Indoor Overnight* | | Outdoor Gathering | Animal Pen |  |
| *An. coustani* s.l. | 8 | 0 | | 14 | 24 | 0 | 0 | | 4 | 3 | 53 |
| *An. funestus* s.l. | 527 | 0 | | 99 | 70 | 19 | 0 | | 0 | 1 | 716 |
| *An. funestus* s.s. | 671 | 468 | | 155 | 116 | 153 | 96 | | 31 | 23 | 1713 |
| *An. gambiae* s.s. | 27 | 10 | | 16 | 4 | 4 | 0 | | 1 | 0 | 62 |
| *An. gibbinsi* | 69 | 4 | | 104 | 265 | 5 | 0 | | 0 | 2 | 449 |
| *An. maculipalpis* | 4 | 1 | | 11 | 85 | 0 | 0 | | 0 | 0 | 101 |
| *An. rufipes* | 1 | 0 | | 0 | 0 | 0 | 0 | | 0 | 0 | 1 |
| *An. sp. 9* | 0 | 0 | | 0 | 1 | 0 | 0 | | 0 | 0 | 1 |
| *An. sp. 15* | 3 | 0 | | 1 | 9 | 0 | 0 | | 0 | 0 | 13 |
| *An. squamosus* | 10 | 1 | | 17 | 118 | 1 | 0 | | 1 | 1 | 149 |
| *An. theileri* | 1 | 0 | | 0 | 0 | 0 | 0 | | 0 | 0 | 1 |
| *An. UG1* | 0 | 0 | | 1 | 3 | 0 | 0 | | 0 | 0 | 4 |
| *An. UG2* | 0 | 0 | | 1 | 0 | 0 | 0 | | 0 | 0 | 1 |
| *Unidentified* | 6 | 1 | | 7 | 33 | 3 | 0 | | 0 | 1 | 51 |
| **Total** | **1327** | **485** | | **426** | **728** | **185** | **96** | | **37** | **31** | **3315** |
| *Overnight collections were only performed indoors for one replicate. | | | | | | | | | | | |
