# Supplementary material for: Early-evening indoor and outdoor foraging by major malaria vectors in Nchelenge, Zambia
Source: PLOS Glob Public Health. 2026 Jul 27;6(7):e0005307. doi: 10.1371/journal.pgph.0005307 (PMC13405103; doi:10.1371/journal.pgph.0005307)
Supplement: S4 Table — *n includes all samples that were molecularly confirmed (excludes 716 An. funestus samples that were not processed). ǂ misidentification calculation excludes samples that were morphologically unidentified. misid = misidentified; misassign. = missassigned (DOCX) [file pgph.0005307.s004.docx]

**S4 Table. Morphological vs. molecular assignment of all molecularly processed specimens.**

| **Molecular ID** | **n*** | **Morphology** | | | | | | | | | | **% misID^ǂ^** |
| --- | --- | --- | --- | --- | --- | --- | --- | --- | --- | --- | --- | --- |
|  |  | *coustani* | *funestus* | *gambiae* | *gibbinsi* | *maculipalpis* | *paludis* | *squamosus* | *tenebrosus* | *zeimanni/ namibiensis* | unidentified |  |
| ***coustani* s.l.** | 53 | **23** | 0 | 0 | 0 | 0 | **12** | 0 | **3** | **2** | 13 | 0.0% |
| ***funestus* s.s*.*** | 1713 | 0 | **1706** | 1 | 5 | 0 | 0 | 0 | 0 | 0 | 1 | 0.3% |
| ***gambiae* s.s.** | 62 | 0 | 0 | **61** | 0 | 0 | 0 | 0 | 0 | 0 | 1 | 0.0% |
| ***gibbinsi*** | 449 | 0 | 5 | 0 | **440** | 0 | 0 | 0 | 0 | 0 | 4 | 1.1% |
| ***maculipalpis*** | 101 | 0 | 0 | 0 | 0 | **90** | 0 | 1 | 0 | 0 | 9 | 1.1% |
| ***squamosus*** | 149 | 0 | 0 | 0 | 0 | 3 | 0 | **123** | 0 | 0 | 22 | 2.4% |
| ***rufipes*** | 1 | 0 | 0 | 0 | 0 | 0 | 0 | 0 | 0 | 0 | 1 | - |
| ***theileri*** | 1 | 0 | 0 | 0 | 1 | 0 | 0 | 0 | 0 | 0 | 0 | 100% |
| ***sp. 9*** | 1 | 0 | 0 | 0 | 1 | 0 | 0 | 0 | 0 | 0 | 0 | 100% |
| ***sp. 15*** | 13 | 0 | 0 | 0 | 0 | 0 | 0 | 10 | 0 | 0 | 3 | 100% |
| ***sp. ug1*** | 4 | 0 | 4 | 0 | 0 | 0 | 0 | 0 | 0 | 0 | 0 | 100% |
| ***sp. ug2*** | 1 | 0 | 0 | 0 | 0 | 0 | 0 | 0 | 0 | 0 | 1 | - |
| **total** | 2548 | 23 | 1715 | 62 | 447 | 93 | 12 | 134 | 3 | 2 | 55 | 1.2% |
| **% misassign.** | 3.4% | 0% | 0.5% | 1.6% | 1.6% | 3.2% | 0% | 8.2% | 0% | 0% |  |  |
| *n includes all samples that were molecularly confirmed (excludes 716 *An. funestus* samples that were not processed) | | | | | | | | | | | | |
| ǂ misidentification calculation excludes samples that were morphologically unidentified | | | | | | | | | | | | |
| misid = misidentified; misassign. = missassigned | | | | | | | | | | | | |
