# Supplementary material for: Early-evening indoor and outdoor foraging by major malaria vectors in Nchelenge, Zambia
Source: PLOS Glob Public Health. 2026 Jul 27;6(7):e0005307. doi: 10.1371/journal.pgph.0005307 (PMC13405103; doi:10.1371/journal.pgph.0005307)
Supplement: S5 Table — * Excluded specimens missing an abdomen. + Indoor overnight trap specimens were only collected for one replicate (DOCX) [file pgph.0005307.s005.docx]

**S5 Table. Visual *An. funestus* blooded abdomen status by trap type.**

| Trap type | | Time | Abdomen status | | | | Total* | % Visually blooded |
| --- | --- | --- | --- | --- | --- | --- | --- | --- |
|  |  |  | No abdomen | Gravid | Visually fed | Visually unfed |  |  |
| Animal pen | 16:00-22:00 | | 4 | 4 | 9 | 193 | 206 | 4.4% |
| Outdoor gathering | 16:00-22:00 | | 7 | 4 | 22 | 253 | 279 | 7.9% |
| Indoor | 16:00-22:00 | | 20 | 42 | 177 | 1128 | 1347 | 13.1 % |
| Indoor^+^ | 22:00-06:00 | | 14 | 2 | 111 | 437 | 550 | 20.2% |
| Total | |  | 45 | 52 | 319 | 2011 | 2382 | 13.4% |
| * Excluded specimens missing an abdomen | | | | | | | | |
| ^+^ Indoor overnight trap specimens were only collected for one replicate | | | | | | | | |
